# Well-Being without a Roof: Examining Well-Being among Unhoused Individuals Using Mixed Methods and Propensity Score Matching

**DOI:** 10.3390/ijerph17197228

**Published:** 2020-10-02

**Authors:** Naina J Ahuja, Allison Nguyen, Sandra J Winter, Mark Freeman, Robert Shi, Patricia Rodriguez Espinosa, Catherine A Heaney

**Affiliations:** 1 Stanford Prevention Research Center, Stanford School of Medicine, Stanford University, Palo Alto, CA 94304, USA; naina.jessica@gmail.com (N.J.A.); altnguyen8@gmail.com (A.N.); swinter@seniorcoastsiders.org (S.J.W.); freemanm@alumni.stanford.edu (M.F.); roberts7@stanford.edu (R.S.); prespinosa@stanford.edu (P.R.E.); 2 Department of Psychology, Stanford University, Stanford, CA 94305, USA

**Keywords:** well-being, unhoused, propensity matching, mixed methodology

## Abstract

The morbidity and mortality experiences of people who are unhoused have been well-described, but much less is known about the overall well-being of these individuals. In this mixed methods study, housed and unhoused participants completed a multi-faceted 10 domain measure of well-being (the Stanford WELL Survey), and a subset of unhoused participants shared their experiences during qualitative interviews. Using propensity score matching, unhoused participants (*n* = 51) were matched at a ratio of 1:5 with housed participants (*n* = 255). The mean overall well-being score of the unhoused participants was significantly lower than that of the matched housed participants (B = −5.022, *p* = 0.013). Additionally, the two groups differed on some of the constituent domains of well-being, with unhoused participants reporting statistically significantly lower mean scores on social connectedness (B = −1.086, *p* = 0.000), lifestyle and daily practices (B = −1.219, *p* = 0.000), stress and resilience (B = −0.493, *p* = 0.023), experience of emotions (B = −0.632, *p* = 0.009), physical health (B = −0.944, *p* = 0.0001), and finances (B = −3.099, *p* = 0.000). The unhoused participants had a statistically significantly higher mean score for spirituality and religiosity (B = 2.401, *p* = 0.000) than their matched housed counterparts. The qualitative interviews further highlighted spirituality and religion as a coping mechanism for the unhoused. The results of this study highlight both unexpected strengths exhibited by the unhoused individuals and areas of challenge.

## 1. Introduction

Homelessness continues to be a pressing social problem in the United States (US) and across the globe. Estimates indicate that approximately 150 million individuals are homeless worldwide, with 20% of the global population being impacted by unstable housing to some degree. In 2018, the US Department of Housing and Urban Development (HUD) reported that 553,742 people experience homelessness on any single night in America [1,2]. The homeless population in the US is very diverse in terms of gender, race, ethnic background, and age [1]. HUD uses the term “homeless” to define someone who lacks a fixed, regular, and adequate night-time residence. In the US, approximately two thirds (65%) of those reported as being homeless were staying in emergency shelters or transitional housing programs [3].

Much research has documented the elevated prevalence of physical and mental health problems among homeless individuals [4]. This increased morbidity contributes to a shorter life expectancy, with reports indicating the average life expectancy of people who experience homelessness to be around 50 years of age [5]. This is approximately 25 years shorter than the general life expectancy of women and men in the US [6]. Poor health is both a cause and a result of homelessness. The causes of homelessness are complex, but often poor physical health, trauma, mental illness, and substance use problems contribute to the downward slide into homelessness [7]. Once homeless, people are at even greater risk for developing or exacerbating these same health problems and developing additional ones, including musculoskeletal disorders, poor oral health, skin and foot problems, and infectious diseases such as tuberculosis, hepatitis C, and HIV [7]. Moreover, during homelessness, many health problems go untreated or are only sporadically addressed due to a lack of access to medical care [8].

While the morbidity and mortality experiences of people who are homeless have been well described, much less is known about the overall well-being of these individuals. Common sense suggests that people who do not have the basic needs of shelter and security met would likely suffer decrements to their overall well-being. Psychological theory also supports this notion. The classic hierarchy of needs model put forth by Maslow suggests that basic physiological and safety needs must be met before other psychological and affiliation needs can be addressed [9]. A more recent motivational stress theory, the conservation of resources (COR) theory [10], also hypothesizes lower well-being among people who are homeless. This theory is based on the notion that people are highly motivated to conserve and enhance their resources—both material resources and psychosocial resources. Resource loss is highly salient and distressing to people, and thus is to be avoided if at all possible. COR theory posits that once a significant resource loss (such as homelessness) has been experienced, it is very hard to stop the downward spiral and reverse course as one needs to invest resources in order to garner further resource gain. Those with greater resources are more capable of orchestrating resource gain, and those with fewer resources are less capable of resource gain. By definition, people who are homeless are lacking important material resources. Therefore, COR theory suggests that they will have great difficulty in garnering future resources (both material and psychosocial) that are necessary for the experience of well-being.

However, other psychological theory hints at pathways to replenishing well-being among people who are homeless. Diener, Lucas, and Scollon discuss theories that rely on the concept of adaptation to changing situations and conditions [11]. Theories such as the hedonic treadmill theory suggest that a person’s well-being is only temporarily affected by these changes, but then returns to an original set point over time [11]. Social production function theory emphasizes the role of substitutability of strategies or pathways for attaining well-being [12]. According to this theory, as specific resources become unavailable (e.g., housing), individuals may emphasize the importance of other types of resources (e.g., belongingness) in their assessments of their own well-being.

The sparse extant research assessing well-being among people who are homeless offers little guidance in terms of which theories most accurately predict the well-being experience of people who are homeless. One study assessed the life satisfaction of homeless individuals in India and the US. Those in the US expressed dissatisfaction with their lives overall. When specific life domains were asked about, they expressed more satisfaction with the self-related domains (e.g., one’s intelligence or one’s self overall) and social domains (e.g., friends or family) than they did with material domains such as income or housing [13,14]. In the United Kingdom, a small qualitative study was conducted with temporarily housed adults to explore their perceptions of well-being. Among these adults, well-being was positively linked with staying busy (e.g., having opportunities for participation in life activities) and maintaining a strong sense of purpose in life. Another study found that a sense of self-confidence was an important aspect of the experience of well-being among individuals who are homeless [15].

A recent mixed methods study based in a Dutch homeless shelter facility emphasized the importance of social participation, defined as “involvement in activities that provides social interaction with others in society or the community” [16]. Results indicated a strong interconnectedness among physical health, psychological health, social participation, taking responsibility for one’s own life, and overall well-being. For example, when a person loses a job for health reasons, they may not only suffer financially but also lose a sense of control over one’s life trajectory, all of which may decrease their overall well-being [17]. Nonetheless, these aforementioned studies only included data from individuals experiencing homelessness; thus, they cannot shed light on whether their findings illustrate unique experiences of people who are homeless or if similar results might be found among people who are housed.

In order to better understand well-being among those who are homeless, our study used a multi-method approach to compare and contrast a sample of individuals in transitional housing with a matched sample of housed individuals in terms of overall well-being and 10 constituent domains of well-being. For the purposes of this study, individuals in transitional housing are referred to as “unhoused”. The research questions include: (1) To what extent are the levels of well-being among unhoused individuals lower than the levels among matched housed individuals? (2) To what extent do unhoused individuals find substitute resources for well-being to replace the resources that they have lost? and (3) How do unhoused individuals qualitatively describe their lived experiences and their contribution to well-being?

## 2. Materials and Methods

This mixed method study was approved by the Stanford University Institutional Review Board (IRB protocol #32814). All individuals provided informed consent, after which they completed an online survey. For the qualitative data collection using semi-structured interviews and focus groups that were audio recorded, an additional consent was obtained that included descriptions of the limits to confidentiality inherent in focus groups. The procedures for the survey portion of the study will be described first, followed by the procedures for the qualitative data collection and analysis.

The unhoused sample (*n* = 51) was recruited via a community partnership with an agency that provides transitional housing and supportive services for homeless families and individuals. To be eligible to live in one of this agency’s shelters, a person must be homeless or facing eviction and currently living or working in the two-county northern California catchment area of the agency. Unhoused participants were recruited at evening sessions held at various shelter locations. The shelter staff advertised the sessions to their clients. During these sessions, individuals were given an opportunity to complete the Stanford WELL Survey.

The sample of 51 unhoused individuals was then matched to housed individuals drawn from the WELL Registry, an online database to which participants were recruited via listservs, social media, and other community partnerships. WELL Registry participants living in California (*n* = 3513) were eligible to be matched with the unhoused sample.

Optimal propensity score matching was used to match the unhoused sample to participants from the WELL Registry on age, gender, education, race, and ethnicity. Propensity scores aggregate multiple potential confounders into a single dimension, which is then used to match individuals from the different groups [18,19,20]. Propensity score matching algorithms provide improved balance in the distribution of covariates across samples or groups, and offer a rigorous way of analyzing observational data containing multiple groups [18,21]. Some unhoused individuals chose not to share some demographic data. We decided to follow the guidance of Stuart (2010), considering missingness as part of the profile of an unhoused participant, so when conducting the match participants were comparably grouped in terms of both covariate values and covariate missingness. An unhoused individual was matched with a housed individual who also did not want to answer specific demographic information about themselves. The R package “Optmatch” (Version 0.9.11) was used to perform the matches without replacement at a ratio of unhoused to housed of 1:5 [19].

**Figure 1:**
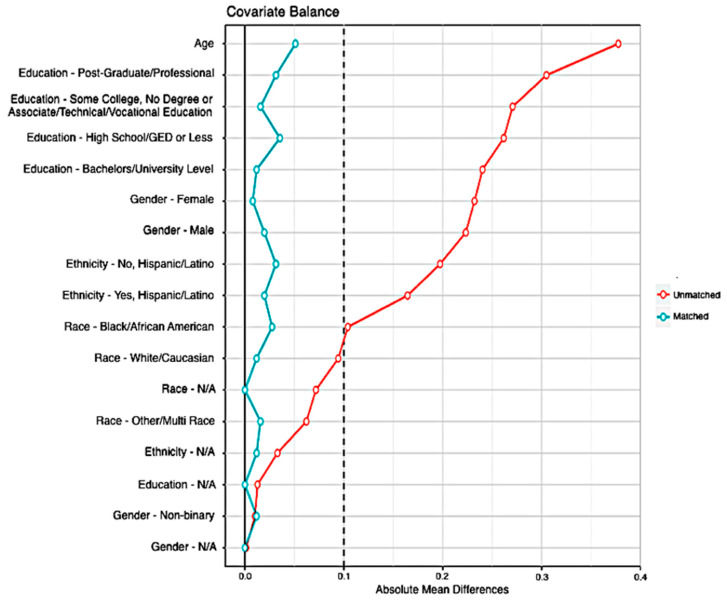
Absolute standard mean differences of unmatched samples and matched samples on the variables included in the development of the propensity scores. The red unmatched sample line indicates the SMDs for the covariates between the full unhoused sample (*n* = 51) and the full eligible WELL housed participant sample (*n* = 3462). Matched samples are the full unhoused sample and the WELL subgroup chosen through propensity score matching (*n* = 255). Categories labeled N/A indicate missing data and were used to allow the matching algorithm to model missing data on that covariate.

Match quality was determined by calculating the absolute standardized mean difference (SMD) between the unhoused sample and the matched housed sample on the matching variables of age, gender, education, race, and ethnicity. A conservative criterion of an SMD < 0.10 for all matched variables was used to indicate a successful match and assessed through the R package “Cobalt” Version 3.7.0 [22]. Figure 1 graphically presents the SMDs between the unhoused and the housed samples both before and after matching. The red line represents the SMDs for the covariates between the unhoused and housed samples before matching, and the blue line represents after matching.

All study participants completed the Stanford WELL Survey. This 76-item questionnaire measures 10 domains of well-being [23]. These domains are presented in Table 1, along with definitions and example questionnaire items. The WELL domains were identified through an innovative measurement development process using narrative inquiry to capture the experiences and perspectives of a diverse set of people. Rigorous coding and analysis of over 100 in-depth interviews resulted in the 10 domains in Table 1 [24].

For each domain, a score from 0–10 was created based on the responses to the constituent items. Higher scores on each domain indicate more optimal levels of well-being. For example, a higher score for the experience of emotions domain indicates more frequent positive emotions and less frequent negative emotions. The domain scores were summed to create the overall well-being score, for a total possible score of 100.

After matching procedures, the survey data were analyzed using linear regression models to estimate differences between the unhoused and the housed individuals in overall well-being and domain-specific well-being. A binary categorical variable (e.g., 1 = unhoused and 0 = housed) was used as the key independent variable representing group differences in the various models. The overall WELL score was the primary dependent variable. Additional models were fit with the constituent domains of the WELL score as the dependent variables. All models adjusted for the matched covariates of age, gender, education, race, and ethnicity as another layer of precaution against biased estimation [18]. All analyses were performed in R version 3.3.3 [25].

The qualitative portion of our study took place after the survey data had been analyzed. Unhoused participants who had completed the Stanford WELL Survey were re-contacted and invited to participate in focus groups or one-on-one interviews to more fully discuss the role of various well-being domains in their lives. The qualitative data collection aimed to: (1) deepen our understanding of the well-being experiences and perspectives of unhoused individuals, and (2) illuminate the meaning of some of the quantitative results from the Stanford WELL Survey. Three focus groups (with a total of 12 participants) and one individual semi-structured interview were conducted.

The interview guide was informed by preliminary analyses of the Stanford WELL Scale data. Questions centered primarily on the domains of stress and resilience, purpose and meaning, and spirituality and religiosity, in order to better interpret the survey findings from these domains. After consent, participants were asked to describe their relevant thoughts, opinions, and experiences about these topics. The discussions were facilitated using strategies based on published best practices [26]. Participants were compensated for their time with gift cards.

All qualitative data were audio recorded and transcribed verbatim using NVivo’s Transcription service [27]. This service uses automated software to transcribe audio recordings, which were then further reviewed and edited by the research team to ensure accuracy. The final transcripts were then coded using an inductive coding approach [28]. Detailed reading of the raw transcripts was used to derive codes [28]. During the coding process, three team members met regularly to discuss new codes and iterations of the coding process. Analytical memos and post-coding analysis were used to further explore associations between codes and to derive potential explanations for some of the survey results. All qualitative data analysis was completed using the NVivo 12 qualitative data analysis software [27].

## 3. Results

### 3.1. Demographics of the Matched Samples

Table 2 summarizes the demographic characteristics of the unhoused sample and the matched housed sample, with expected similarities in the demographic composition of the two samples after matching. About half of each sample was female. The educational attainment of the participants, with about three quarters not having attained a bachelor’s degree, is low by local standards. Approximately a quarter of the participants self-identified as Hispanic.

### 3.2. Survey Results Comparing Well-Being Between the Unhoused and Housed

Table 3 presents the regression coefficients for the binary categorical variable (1 = unhoused, 0 = housed) predicting overall well-being and the constituent well-being domains. For overall well-being, the unhoused participants scored, on average, 5.022 points lower than did their matched housed counterparts (*p* = 0.013). Additionally, the two groups significantly differed on some of the constituent domains of well-being. The domain scores for social connectedness (B = −1.086; *p* < 0.001), lifestyle and daily practices (B = −1.219; *p* = 0.000), stress and resilience (B = −0.493; *p* = 0.023), experience of emotions (B = −0.632; *p* = 0.009), physical health (B = −0.944; *p* < 0.001), and finances (B = −3.099; *p* < 0.000) were lower among the unhoused compared to their housed counterparts.

Some of the differences cited above were due to specific sub-domains. Within the lifestyle and daily practices domain, people in the unhoused group were less likely to experience high-quality sleep (B = −1.292; *p* = 0.000) and have a high-quality diet (B = −1.169; *p* = 0.000). Within the experience of emotions domain, while differences did not emerge for positive emotions, the unhoused group reported experiencing more frequent negative emotions. Within the stress and resilience domain, people in the unhoused group did not report experiencing higher levels of stress than did those in the housed group, but they did report lower levels of resilience.

In only one domain (spirituality and religiosity), people who were unhoused exhibited higher scores than those who were housed (B = 2.401; *p* < 0.001). A few constituent domains of well-being showed no significant differences between the unhoused and housed participants. The domain scores for purpose and meaning, sense of self, and exploration and creativity did not differ between the groups.

### 3.3. Qualitative Results

The sub-sample of unhoused individuals who participated in the qualitative data collection provided rich insights into their personal experiences of well-being. The average age for this sample (*n* = 13) was 54, ranging from 33 to 70 years of age. Participants were predominantly male (69%), white (69%), and non-Hispanic (92%). Only one participant was born outside of the US. There was variability in educational attainment, with 33% of participants having attained a high school level education or less, 50% having attended some college, and 17% having attained bachelor level degrees. The majority of the participants were single (62%), followed by married participants (23%), and a few divorced or widowed (15%). Nearly half of the participants (46%) had children. Note that the demographic composition of the participants in the qualitative sub-study differed from that of the sample of unhoused participants who completed the survey. Thus, insights gained from the qualitative data should be construed as exploratory and may not represent the experience of the unhoused sample overall. Insights are described below, focused on the domains of spirituality and religiosity, experience of stress, and purpose and meaning in life.

#### 3.3.1. Reliance on Spirituality and Religiosity

Several unhoused participants suggested that a reliance on religion serves a key role in helping unhoused individuals cope with their circumstances. Participants described the day-to-day struggles and incredible difficulties associated with housing instability, and stated how seeking help from religion was important, particularly when support or help from other sources was limited. One participant stated, “You don’t have any family, you don’t have a lot of connections, you reach out to see if something else works for you” (male participant). Another male participant said, “When we feel alone we want something to hold onto, to grasp”, and contrasted this with the experience of housed individuals: “When life is good … you’re not asking God for anything, you’re not even thanking him, probably.”

For some, reliance on religion was a last resort. One male participant explained that, “When you’re at the bottom, you’re reaching for something, and the last thing you can reach for is your religion or your spirituality.” Another male participant compared the seeking of spirituality and religiosity as an unhoused individual to the coping strategies someone experiencing incarceration might engage in: “I mean like when everyone goes to jail, what’s the first thing they do? Everybody gets God.” Participants elaborated that although drugs and alcohol could also serve as coping mechanisms, unlike spirituality and religiosity, drugs and alcohol were viewed as temporary relief. Several of the participants who had either turned to drugs and alcohol or witnessed it as a coping strategy among peers stated that religion was an answer for them when drugs and alcohol were no longer able to meet that need.

#### 3.3.2. Stress Experiences

Focus group members were not surprised by the survey results showing no statistically significant difference in stress levels between the unhoused and matched housed participants. They suggested that despite the many stressors experienced by those who are unhoused, housed individuals also have many stressors that may account for the two groups’ similar overall levels of stress. Some of the stressors of those who are housed were characterized as the “hustling and bustling … worrying about paying bills, taking care of yourself, taking care of your kids and everything”, and “bills, obligations that are financial.” For one male participant, he felt that “it wasn’t until I lost everything that I could relax.” The high cost of living in the local area was cited as a potential key reason for added stress for housed participants. Several participants made comments similar to the following:

“Well it’s so expensive to live here, there’s so much traffic. Unless you’re retired and have a bunch of money, costs you three thousand dollars to get a studio [apartment]. It’s ridiculous. You got to work your butt off to get by. There’s a lot of stress there. And your quality of life. You might be working fifteen hours a day to get there.”

Demands and responsibilities related to families were also cited as potential key stressors for housed participants: “If you have children involved and you’re having to get up and cook for them and clean for them and take them to school, that might be different you know, because helping a household.” For other participants, the presence of stress itself was seen as universal regardless of housing status, but that the form it takes can be different among different people. One participant said, “I don’t think it makes much of a difference that you [have] stable housing, you still have other problems to deal with as well. You just realize there are just a different set of problems that require different solutions to.”

#### 3.3.3. Purpose and Meaning Experiences

Discussions around purpose and meaning among unhoused individuals were intrinsically tied to self-exploration and motivations to improve their current circumstances. Several participants found that being unhoused allowed them to seriously reflect on how they came to be in this situation. A female participant explained that being unhoused “should make you look more inside yourself … and really figure out. Because you’re going through something for a reason, whether it’s bad or good.”

This reflection led some participants to consider factors such as their upbringing or their social support networks, and how these factors played a role in their path to becoming unhoused. For example, one female participant explained that she became unhoused “because the landlord didn’t like the other person that was there.” Another female participant explained that she was “taking care of [her] mom and [her] daughter and [her] mom died, and the housing went with her … it wasn’t [her] choice, it was just something that happened.” These kinds of external factors played a role in shaping their belief systems about purpose in life. A male participant elaborated, “Everything that we did in our pasts that put us exactly where we are now, prepares us for what’s next. […] Go out there and find what you’re here to do, which is a trip.”

For many participants, their purpose was tied to improving their current housing situation. This motivation was primarily driven by either finding stable housing or returning to a stable family or housing situation they had lost: “My motivation is to get out of here, get a house, get back on my feet again, to get back into my family’s life again” (male participant). Some participants also reflected on how their purpose and motivation had changed across various periods of housing instability. A male participant who was living in transitional housing for a second time stated:

“The first time that I came here, I sat on my butt for six months, didn’t try to work anything, didn’t try to get into a program. [This time] the second I got here, I told myself ‘I’m getting out of here.’ And I was going to do whatever it took to get the [explicit language] up out of here.”

Though most of the participants agreed with the notion that levels of purpose and meaning for those who are unhoused could be similar to the levels experienced by housed individuals, some pointed out that this was not necessarily true at all points in their lives. For example, one female participant described herself as being newly unhoused and found that she had very little sense of purpose—she had no home, no job, and no clean clothes. She explained that perhaps she could see this perspective changing, especially for someone who has been unhoused for longer and was no longer experiencing the shock of being newly unhoused. Therefore, it is important to note that the experience of purpose and meaning may change depending on how long and how frequently a person has been unhoused.

## 4. Discussion

This study explored the differences in overall well-being and domain-specific well-being between unhoused individuals and a matched sample of housed individuals. As expected, the unhoused individuals reported lower levels of overall well-being, as well as lower scores on some constituent domains, including those that could be construed as indicating Maslow’s basic physiological and safety needs (i.e., finances and physical health). Since Maslow’s classic hierarchy of needs model states that basic needs must be met before other higher level needs can be addressed, it is not surprising that the unhoused also reported lower levels of well-being in some of the domains that are more psychological (i.e., experience of emotions and resilience). The qualitative data further contextualized and illuminated the differences (or lack of differences) between unhoused and housed individuals.

Contrary to the findings of Biswas-Diener and Diener, this study found that social connectedness was lower among the unhoused individuals as compared to the housed individuals [13]. A lower social connectedness score reflects having less access to social support and companionship, feeling more socially isolated and lonely, and having a hard time meeting social expectations. The qualitative data also highlighted the lack of social connections experienced by the unhoused participants, feeling isolated from family and other key people in their lives. Lack of social connection is a well-known predictor of morbidity and mortality [25]; thus, intervention in this area should be a key priority moving forward.

Similar to the findings of Biswas-Diener and Diener, sense of self scores were not significantly different from those of the matched housed group [13]. These scores reflect the extent to which people are accepting of themselves and feel good about themselves. In addition, the unhoused individuals expressed a similar level of purpose and meaning in their lives to the housed individuals. These findings do not support Maslow’s framework, where basic needs must be met before psychological needs. Instead, while the unhoused people in our study expressed clear deficits in their basic needs, these results also demonstrated a certain steadfastness in terms of some psychological resources.

To a certain extent, our findings do align with the social production function theory that underscores the importance of substitutability of strategies or pathways for attaining well-being. For example, participants reported substituting religion for other coping strategies that were no longer available to them or were no longer working (e.g., substance use). While levels of purpose and meaning may be similar to those reported by housed individuals, the aspirations and qualities of life that provide purpose and meaning to unhoused individuals may be quite different. The same can be said of stress levels and the nature of stressors. People adapted to the changes in the challenges that they faced and the resources that they had available. However, the lower self-reported levels of resilience among those who are unhoused suggests that there are likely limits to the substitutability function.

Attention should be paid to the external validity of our findings. As of January 2019, approximately 27% of people experiencing any type of homelessness in the United States were located in California, where our study sample was based [29]. The unhoused population in California is predominantly male (55.3%), white (58.15%), non-Hispanic (64.1%), and over the age of 24 (58.5%) [30]. Similarly, our unhoused study sample also skewed to be mostly white (47.1%) and non-Hispanic (68.6%), but was evenly split between males and females. The existing research on the association between sex and well-being in unhoused individuals suggests that women may be a particularly vulnerable subgroup [31]. Unhoused women are less satisfied with their health and empowerment, have lower self-esteem, and experience more psychological distress than unhoused men [32]. Additionally, although findings are mixed as to the degree of vulnerability, the risks of homelessness associated with health and well-being may be greater for people of color than for white people, especially for women [33]. Age also plays a role in the experience of homelessness: older age among unhoused individuals has been associated with worse physical health and more limited social support [31,34]. Thus, the demographics of a homeless individual may influence their experience of homelessness and how they report their well-being. The lower levels of overall well-being as well as the lower scores on constituent domains, such as social connectedness and physical health, between the unhoused and housed individuals found in this study might be more pronounced if the participants included more females, people of color, and older participants.

The results of this study offer several implications for how best to promote the well-being of unhoused individuals. First, the well-being domains that showed clear deficiencies should be addressed. Transitional housing and other services often focus on meeting basic needs such as providing shelter, nourishment, a safe environment, and health care. While this is tremendously important, our study suggests other important targets for intervention. For example, to alleviate feelings of loneliness and low levels of perceived social support, opportunities for developing new social relationships and for helping people maintain or enhance existing relationships should be created. Our study also suggests that unhoused people, even when in transitional housing, still suffer a dearth in the quality of their sleep and their diets. To improve these lifestyle behaviors, focusing on direct, personalized care that can be incorporated into the existing infrastructure of transitional housing or other services for unhoused individuals can be implemented. For example, on-site cooking classes compatible with existing shelter schedules, providing fresh fruit and vegetables, and providing suggestions and materials for good sleep hygiene might be helpful [26].

Second, services for people who are in transitional housing should build upon the strengths exhibited by the unhoused individuals in this study and encourage substitutability. For example, since the unhoused individuals found spirituality and religiosity to be a helpful coping resource, perhaps a cleric could be made available to those in transitional housing (similar to how hospitals provide pastoral care through hospital chaplains) [27]. Clerics and other counselling professionals might also aid in the identification of new aspirations for maintaining purpose in life and staving off feelings of hopelessness or helplessness.

This study explored well-being among people who are unhoused, a nascent but important research arena. While the small non-representative sample of people living in transitional housing limits the generalizability of our findings, the study does provide new hypotheses for further testing. However, it is important to remember that the homeless population is composed of those who are living on the streets, living in emergency shelters, those staying with friends and family on an unstable basis, or living in transitional housing. Our study sample only included the latter group, and thus our results are only applicable to those in transitional housing. This population likely differs in meaningful ways from those who are living on the streets [35,36].

Nonetheless, aspects of our study offered unique opportunities. A robust, multifaceted measure of well-being allowed us to explore both overall well-being and specific constituent domains of well-being in a population that is often understudied. The use of propensity score matching to develop a 1:5 unhoused to housed match strengthened our ability to control for the confounding effects of other demographics, over and above the typical strategy of solely entering the demographics as covariates in traditional regression models. Incorporating the comparison between housed and unhoused individuals allowed us to explore patterns of well-being domains that might be specific to people who are unhoused.

## 5. Conclusions

The present study contributes to the literature on well-being in homeless populations. Participants who were unhoused had overall lower well-being scores than did their matched housed counterparts. Our nuanced measure of well-being provided us the opportunity to hone in on specific domain differences that can inform targeted interventions to improve the quality of life of the unhoused. The potential to strengthen certain well-being domains as substitutes for diminished resources in other areas is a possible path worth exploring for future interventions aimed at improving the lives of homeless populations, a worthy goal in the fight for equality and social justice.

## Figures and Tables

**Table 1:** Definitions of the 10 constituent domains of well-being measured in the Stanford WELL Survey with example items.

Constituent Domain of Well-Being	Definition	Example Items
Social Connectedness	Positive or negative relationships with others and how they influence your well-being	During the last two weeks, how often did you feel…
		1. … that you lacked companionship?
		2. … that there were people you could talk to?
		3. … that you were a part of a group of friends?
Lifestyle and Daily Practices	Lifestyle behaviors that can influence your well-being such as: diet; physical activity; sleep; the use of tobacco, alcohol, and marijuana; and other ways people take care of themselves	1. During the past two weeks, how would you rate your sleep quality overall?
Stress and Resilience	Stress: Feelings of overload and an inability to balance or manage tasksResilience: Ability to adapt to change and bounce back after hardship	1. During the last two weeks, how often have you felt that you were not able to give enough time to the important things in your life?
		2. How confident are you that you can bounce back quickly after hard times?
Experience of Emotions	How often you experience both pleasant and unpleasant emotions	During the last two weeks, how often did you feel…
		1. …calm?
		2. …drained?
Physical Health	Perception of your own health status, i.e., energy levels, ability to resist illness, physical fitness, and experience of pain.	1. Compared to others of your own age, how would you rate your health?
		2. During the last two weeks, how often did your energy level allow you to do the things you WANT to do, as opposed to only the things you have to do?
Purpose and Meaning	Having a sense that aspects of your life provide purpose and meaning, i.e., goals, dreams, and being part of something larger than yourself.	How often does your daily life include experiences that give your life…
		1. … purpose?
		2. … meaning?
Sense of Self	The extent to which you feel you know yourself, can express your true self, have self-confidence, and feel good about who you are.	During the last two weeks, how often did you feel…
		1. … accepting of yourself?
		2. … that you were interested in your daily activities?
Finances	Your perception of having enough money to meet your needs.	1. During the last year, how often have you had enough money to meet your needs?
Spirituality and Religiosity	The extent to which spiritual and religious beliefs, practices, communities, and traditions are important in your life.	1. How important are spiritual or religious beliefs in your day to day life?
Exploration and Creativity	Having opportunities to grow as a person and to explore new experiences and ways of thinking.	1. How often do you engage with opportunities to challenge yourself and grow as a person?

Note. Further details about the Stanford WELL Scale are available from the authors.

**Table 2:** Demographics of the matched housed and unhoused samples.

		Housed (N = 255)		Unhoused (N = 51)	
Variable		N	%	N	%
Age (Mean SD)		46.00 (19.0)		46.82 (13.0)	
Gender	Female	127	49.8	25	49.0
	Male	120	47.1	25	49.0
	Non-Binary	8	3.1	1	2.0
Education	High School/GED or less	71	27.8	16	31.4
	Associate, Some College, No Degree	114	44.7	22	43.1
	Bachelors/University Level	32	12.5	7	13.7
	Post-Graduate/Professional	33	12.9	5	9.8
	Missing	5	2.0	1	2.0
Race	White/Caucasian	117	45.9	24	47.1
	Black/African American	42	16.5	7	13.7
	Other/Multi Race	36	14.1	8	15.7
	Missing	60	23.5	12	23.5
Ethnicity	Hispanic	65	25.5	14	27.5
	Not Hispanic	183	71.8	35	68.6
	Missing	7	2.7	2	3.9

**Table 3:** Regression coefficients for being unhoused when predicting overall well-being score and the scores of the 10 constituent domains of well-being.

Domain	Regression Coefficient (SD) ^1^
Overall Well-being	−5.022 * (1.989)
Social Connectedness	−1.086 *** (0.251)
Lifestyle and Daily Practices	−1.219 *** (0.188)
Diet	−1.169 *** (0.239)
Physical Activity	−0.662 (0.452)
Sleep	−1.292 *** (0.295)
Stress and Resilience	−0.493 * (0.214)
Stress	−0.293 (0.260)
Resilience	−0.692 ** (0.238)
Experience of Emotions	−0.632 ** (0.240)
Positive Emotions	−0.521 (0.268)
Negative Emotions	−0.742 ** (0.263)
Sense of Self	−0.395 (0.290)
Purpose and Meaning	0.149 (0.323)
Physical Health	−0.944 *** (0.238)
Finances	−3.099 *** (0.457)
Spirituality and Religiosity	2.401 *** (0.507)
Exploration and Creativity	0.038 (0.341)

^1^ All regression models included age, gender, race/ethnicity, and education as covariates. Reference group is housed participants. * *p* value < 0.05, ** *p* value < 0.01, *** *p* value < 0.001.
